# A Potent Malaria Transmission Blocking Vaccine Based on Codon Harmonized Full Length *Pfs48/45* Expressed in *Escherichia coli*

**DOI:** 10.1371/journal.pone.0006352

**Published:** 2009-07-22

**Authors:** Debabani Roy Chowdhury, Evelina Angov, Thomas Kariuki, Nirbhay Kumar

**Affiliations:** 1 Department of Molecular Microbiology and Immunology, Johns Hopkins University, Bloomberg School of Public Health, Baltimore, Maryland, United States of America; 2 U.S. Military Malaria Vaccine Program, Walter Reed Army Institute of Research- Naval Medical Research Center (WRAIR-NMRC), Division of Malaria Vaccine Development, Silver Spring, Maryland, United States of America; 3 Institute of Primate Research, National Museums of Kenya, Nairobi, Kenya; London School of Hygiene & Tropical Medicine, United Kingdom

## Abstract

Malaria caused by *Plasmodium falciparum* is responsible for nearly 1 million deaths annually. Although much progress has been made in the recent past, the development of a safe, effective and affordable malaria vaccine has remained a challenge. A vaccine targeting sexual stages of the parasite will not only reduce malaria transmission by female *Anopheles* mosquitoes, but also reduce the spread of parasites able to evade immunity elicited by vaccines targeting pre-erythrocytic and erythrocytic asexual stages. We focused our studies on Pfs48/45, a protein expressed in the sexual stages developing within an infected person and one of the most promising transmission-blocking vaccine targets. Functional immunogenicity of Pfs48/45 protein requires proper disulfide bond formation, consequently evaluation of the immunogenicity of recombinant full-length Pfs48/45 has been hampered by difficulties in expressing properly folded protein to date. Here we present a strategy involving harmonization of codons for successful recombinant expression of full length Pfs48/45 in *Escherichia coli*. The purified protein, designated CH-rPfs48/45, was recognized by monoclonal antibodies directed against reduction-sensitive conformational epitopes in the native protein. Immunogenicity evaluation in mice revealed potent transmission blocking activity in membrane feeding assays of antisera elicited by CH-rPfs48/45 formulated in three different adjuvants, i.e. Alum, Montanide ISA-51 and complete Freund's adjuvant. More importantly, CH-rPfs48/45 formulated with Montanide ISA-51 when administered to nonhuman primates (Olive baboons, *Papio anubis*) resulted in uniformly high antibody responses (ELISA titers >2 million) in all five animals. Sera from these animals displayed greater than 93% blocking activity in membrane feeding assays after a single immunization, reaching nearly complete blocking after a booster dose of the vaccine. The relative ease of expression and induction of potent transmission blocking antibodies in mice and nonhuman primates provide a compelling rationale and basis for development of a CH-rPfs48/45 based malaria transmission blocking vaccine.

## Introduction

Based on most recent estimates from the WHO, worldwide, there were an estimated 247 million malaria cases among 3.3 billion people at risk living in 109 countries [1]. Infections caused by *P. falciparum* and *P. vivax* account for more than 90% of global malaria burden; the former being responsible for nearly all the deaths due to malaria, nearly a million deaths of children under 5 years [2]. Some of the current efforts against malaria include increasing use of insecticide treated bed nets and use of combination drugs to tackle the problem associated with drug resistance [3], [4]. The emergence of drug-resistant strains over the last 4 decades has emphasized the necessity of new control strategies. In this regard, the development of a safe and effective malaria vaccine is expected to play important and critical role in controlling malaria [5]. Such vaccine development efforts have focused on candidate antigens represented in the pre-erythrocytic, erythrocytic and sexual stages of the parasite. Currently, the only vaccine advanced in clinical development, RTS,S, has shown partial protection against infection and disease severity in several clinical trials [6], [7].

Immunity against the sexual stages of the parasite offers an effective way to reduce or stop malaria transmission and in that respect offers the greatest promise towards the goal of progressively eliminating malaria from endemic countries. A transmission blocking vaccine (TBV) [8] specifically targeting the sexual development of the parasite in the mosquito vector may elicit immunity which can effectively block transmission of the parasite from invertebrate mosquito vector to vertebrate host. Transmission of malaria depends upon the presence of infectious male and female gametocytes in the peripheral blood of infected persons and successful ingestion of these gametocytes by *Anopheles* mosquitoes. Soon after ingestion, exflagellation occurs within the mosquito midgut, and emergent male gametes fertilize female gametes, resulting in the formation of zygotes. The zygotes undergo post-fertilization transformation into motile ookinetes which traverse the midgut epithelium and develop into oocysts resulting in the production of infective sporozoites. Finally the sporozoites are released into the hemocoel, invade the salivary glands and are transmitted to vertebrate hosts during subsequent blood feeding [9].

The targets of transmission blocking antibodies include pre-fertilization antigens (Pfs230 and Pfs48/45) expressed in the circulating gametocytes and post-fertilization antigens (Pfs25 and Pfs28) expressed during mosquito stage ookinete development [10]. Unlike Pfs25 and Pfs28, pre-fertilization antigens are also targets of the natural immune response and thus immunity induced by a vaccine based on any of these antigens will have the added benefit of natural boosting of immunity. To date, only Pfs25 and Pvs25 (*P. vivax* homolog of Pfs25) have undergone limited Phase I clinical trials with marginal success [11], [12]. So far it has not been possible to evaluate any of the pre-fertilization antigens as vaccines simply because they have not been available in sufficient quantity and proper protein conformation.

Our choice of pre-fertilization antigen Pfs48/45 (encoded by a 1347 bp single open reading frame) over Pfs230 (encoded by a much larger 9.4 kb ORF) was based primarily on the size of the recombinant protein to be expressed. Both are cysteine-rich polypeptides (16 residues in Pfs48/45 and 70 in Pfs230) and due to the conformational nature of target epitopes it has not been possible to further delineate functional protein domains for vaccine development [13]. Additionally, targeted gene disruption studies have shown that Pfs48/45 plays a critical role in male gamete fertility, an important aspect of the sexual reproduction success of the parasite [14]. Analysis of immune human sera in endemic areas has also suggested a strong correlation between naturally present anti-Pfs48/45 antibodies and transmission reducing activity of those human sera; thus making it a key candidate for vaccine development [15]. However, efforts to produce full length recombinant Pfs48/45 in a functional conformation have largely remained unsuccessful. In a recent study, an approach that involved co-expression of a truncated version of C-terminal fragment of Pfs48/45 fused with a large fusion partner maltose binding protein (MBP) along with four periplasmic folding catalysts DsbA, DsbC, FkpA and SurA resulted in correctly folded truncated product which produced high titers of transmission-reducing antibodies in immunized BALB/c mice [16]. In this recombinant expression approach periplasmic targeting and folding into functional conformation of expressed Pfs48/45 protein was strictly dependent upon fusion with MBP as a carrier protein and protein folding was catalyzed by four chaperons co-expressed in the host, respectively.

In this study, we employed an approach that harmonizes codon usage frequency of the target gene with those of the expression host for heterologous expression of protein. Basic studies on regulation of protein expression have shown that synonymous codon substitutions from infrequent to frequent usage in regions where mRNA translation occurs relatively slowly can be detrimental to protein expression and stability [17]. On the other hand, codon substitutions introducing rare codons in the regions containing high frequency codons can lead to erroneous protein conformation [18]. Taking these concepts into account, an algorithm termed “codon harmonization” [19] was developed where synonymous codons from *E. coli* were selected that closely resemble the codon usage of native Pfs48/45 gene, including regions coding ‘link/end’ segments of proteins in *P. falciparum*. This approach harmoniously mimics the translation rate of protein expression in native host by allowing the translation machinery to pause at exactly the same positions in *E. coli* as in *P. falciparum* and as presented in this article yielded expression of correctly folded Pfs48/45 in *E.coli*. The method of codon harmonization has already proved to be successful in facilitating production of several GMP grade malaria vaccines for ongoing vaccine trials [20], [21]. This study reports for the first time the efficient and successful expression of full length TBV candidate Pfs48/45 in high yields and appropriate conformation. The recombinant Pfs48/45 elicits potent malaria transmission blocking antibodies in mice and non human primates (Olive baboons, *Papio anubis*) and suggests a malaria TBV could be developed based on the CH-rPfs48/45 antigen.

## Results

### Expression and purification of correctly folded Pfs48/45

**Figure 1:**
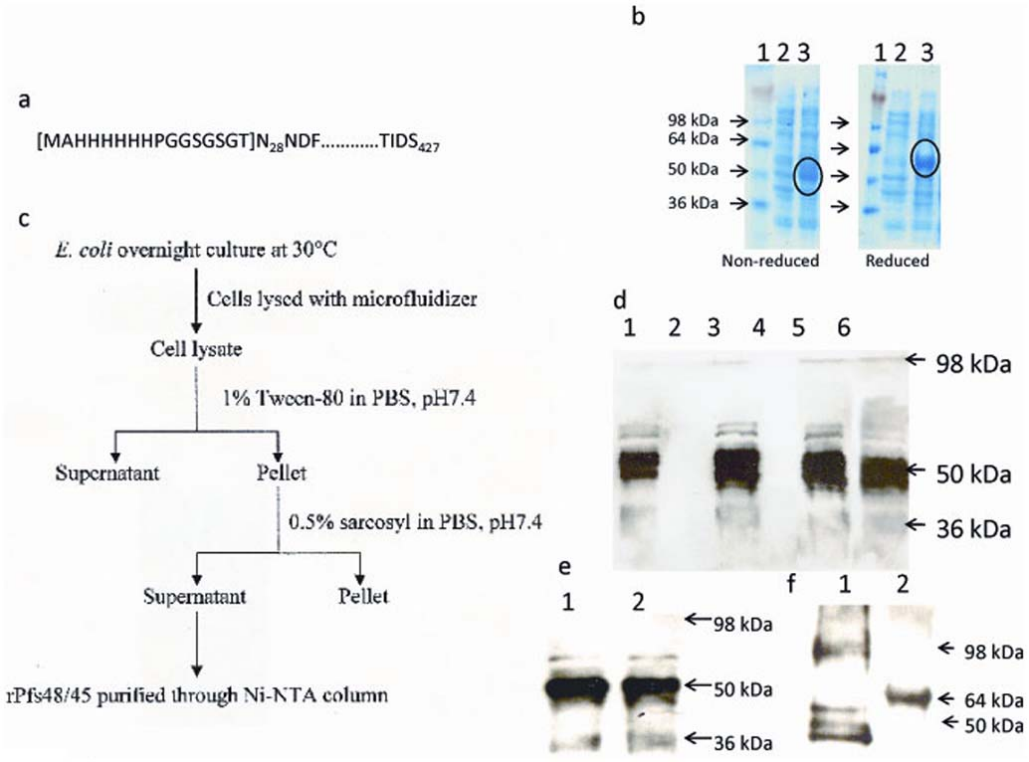
Purification and characterization of CH-rPfs48/45. (a) Schematic representation of amino acid residues in the recombinant protein expressed. First 16 amino residues at the N-terminus contain 6 x Histidine residues and a short linker to allow affinity purification of the protein. The sequence of Pfs48/45 begins with asparagine (N) at the 28 position and ends with serine (S) at 427 position in the native Pfs48/45 sequence. (b) Induction profile of CH-rPfs48/45. The cells were induced with 0.1 mM IPTG for 3 h and lysed by sonication. Lysates of uninduced and induced cells either non-reduced (left panel), or reduced (treated with 10 mM 2-mercaptoethanol, right panel) were run on SDS-polyacrylamide gel and stained with Coomassie stain. Lane 1; Protein molecular weight marker; lane 2, uninduced cell lysate; lane 3, induced cell lysate. The induced protein band in both non-reduced and reduced gels are encircled for easy recognition. (c) Flow diagram of various major steps including differential detergent extractions used for protein purification. (d) Western blot analysis for the presence of CH-rPfs48/45 at each step of detergent extraction using anti-His mAb. Lane 1, lysate pellet; lane 2, lysate supernatant; lane 3, 1% Tween-80 pellet; lane 4, 1% Tween-80 supernatant; lane 5, 0.5% sarcosyl pellet; lane 6, 0.5% sarcosyl supernatant. (e) Western blot analysis of purified CH-rPfs48/45 using anti-His mAb. Lane 1, eluate from Ni-NTA column; lane 2, dialyzed CH-rPfs48/45. (f) Recognition of CH-rPfs48/45 by conformation specific mAb IIC5B10. Lane 1, non-reduced CH-rPfs48/45; lane 2, reduced CH-rPfs48/45.

The native sequence of Pfs48/45 (accession number AF356146) lacking N-terminal signal sequence (amino acid residues 1–27) and C-terminal anchor (amino acid residues 428–448) was converted to ‘codon harmonized’ sequence designed for expression in *E. coli*. The amino acid sequence reported under AF356146 differs from that of Z22145 (NF54) by C32Y, K33N, T72N, K253N and N254K substitutions. The A/T contents of Pfs48/45 sequence before and after codon harmonization were 75% and 56%, respectively. The codon harmonized Pfs48/45 sequence containing a sequence tag coding for 6 x histidines at the 5′ end was synthesized by Retrogen Inc., cloned into the pET (K^−^) expression vector [19] and expressed in BL21 (DE3) cells [Fig. 1a]. Initial attempts to induce the cells with IPTG indicated that the expressed protein might negatively impact the growth of *E.coli*. To overcome the toxicity of expressed protein, we modified expression strategy to slow down protein expression by growing the cells at 30°C in Luria-Bertani growth medium containing 1% glucose and induction with 0.1 mM IPTG for 3 h, which resulted in highly efficient induction of protein in the cell lysate at 50 kDa [Fig. 1b, left panel, encircled]. The cell lysate when treated with β-ME to reduce the disulphide bonds in the protein, showed slower electrophoretic mobility of the induced Pfs48/45 protein [Fig. 1b, right panel, encircled]. A similar observation was made with the native form of the protein on the gametocyte surface [22], [23].

Western blot analysis of cell lysates either untreated or after treatment with 0.5% sarcosyl revealed that the recombinant protein was insoluble in the absence of sarcosyl detergent (data not shown), and hence the treatment with ionic detergent was required to facilitate extraction of the protein from the pellet. To selectively enrich expressed Pfs48/45, the lysate was first treated with non-ionic detergent Tween-80 to remove any soluble bacterial proteins followed by treatment with 0.5% sarcosyl in PBS [Fig. 1c]. This sequential detergent extraction resulted in partial solubilization of the expressed protein which could be further purified using Ni^2+^-NTA-Agarose beads (QIAGEN) by elution using imidazole (1 M), yielding ∼3 mg purified protein/g of wet cell pellet [Fig. 1d, e]. The yield of purified CH-rPfs48/45 in 7 independent purifications varied between 15 and 25 mg per liter of induced culture. The conformational characterization of the expressed protein designated CH-rPfs48/45 was achieved using a mAb IIC5-B10 [22], which recognizes a conformational reduction-sensitive epitope in the parasite derived native Pfs48/45. The mAb recognized the non-reduced form of CH-rPfs48/45 yielding 3 immunoreactive bands at ∼50 kDa of the gel [Fig. 1f, lane 1]. In addition to these monomeric forms, purified CH-rPfs48/45 protein preparations consistently revealed the presence of a higher molecular weight band at ∼98 kDa, presumed to be a dimer. When a similar Western blot analysis was carried out with CH-rPfs48/45 after reduction prior to SDS-PAGE, the mAb rather unpredictably showed recognition of a single band around 65–68 kDa [Fig. 1f, lane 2]. Previous biosynthetic metabolic labeling studies had established that non-reduced Pfs48/45 immunoprecipitated by the mAb IIC5B10 (a doublet migrating in the region of 45 and 48 kDa) coalesces into a single protein band of ∼68 kDa when analyzed by SDS-PAGE after reduction [23]. On the other hand, the same mAb strictly recognized only the non-reduced form of native Pfs48/45 in the gametocytes and gametes in Western blot analysis [24]. While the reasons for the totally unexpected recognition of reduced form of CH-rPfs48/45 are not so obvious, a possible interpretation may be that the functional target epitope of blocking antibodies might be conformationally more stable in the CH-rPfs48/45, and therefore not affected by the reduction conditions employed.

### Evaluation of functional immunogenicity of CH-rPfs48/45 in mice in different adjuvant formulations

**Figure 2:**
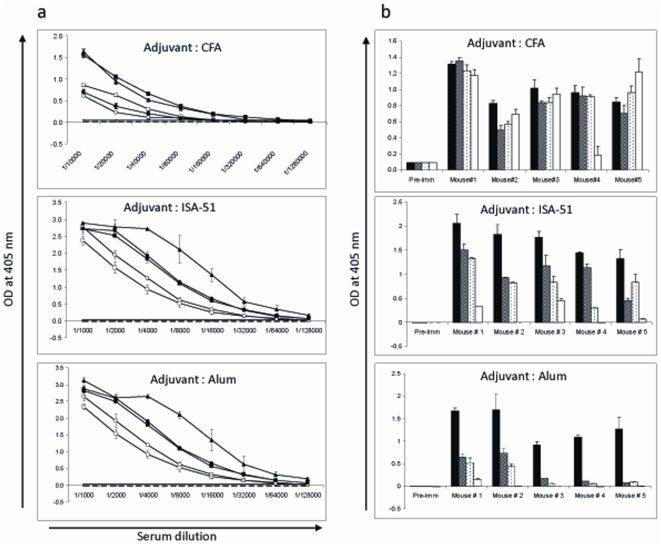
(a) ELISA analysis of CH-rPfs48/45 immunized individual mouse sera in three different adjuvant formulations: Complete Freund's adjuvant (top panel), Montanide ISA-51 (middle panel), and Alum (bottom panel). All the results are representative of three independent experiments. Pooled pre-immune sera + 3SD are shown by broken lines. ELISA OD_405_ values for individual mice are shown; mouse 1 (filled triangle), mouse 2 (open square), mouse 3 (filled square), mouse 4 (open circle), mouse 5 (filled circle). (b) Analysis of anti-Pfs48/45 IgG isotype distribution in individual mouse sera: IgG1 (filled columns), IgG2a (hatched columns), IgG2b (stippled columns), IgG3 (blank columns).

The immunogenicity of CH-rPfs48/45 was assessed in three different adjuvant formulations: CFA, water-in-oil emulsion using Montanide ISA-51 and Aluminium hydroxide. Figure 2a shows the IgG titer 2 weeks after the 2^nd^ boost in CFA group and 2 weeks after the 3^rd^ boost in Alum and Montanide ISA-51 groups. Although CFA is unsuitable for human vaccines, this formulation resulted in the highest antibody titer ranging from 3×10^5^ to >1×10^6^ [Fig. 2a, top panel]. However, all mice immunized with CH-rPfs48/45 in the other two adjuvant formulations also showed high antibody titer, the range being ∼70,000 to more than 100,000 in both Montanide ISA-51 and Alum formulations [Fig. 2a, middle and bottom panels]. We also tested sera for IgG isotype distribution and all four subtypes (IgG1, IgG2a, IgG2b, IgG3) were more or less equally represented in sera from mice immunized with CFA formulation, [Fig. 2b, top panel]. On the other hand, IgG1 and to a lesser extent IgG2 were the dominant subtype in Montanide ISA-51 formulation [Fig. 2b, middle panel]. The overwhelming presence of IgG1 and negligible amount of IgG3 were noticed in the Alum formulation [Fig. 2b, bottom panel].

**Figure 3:**
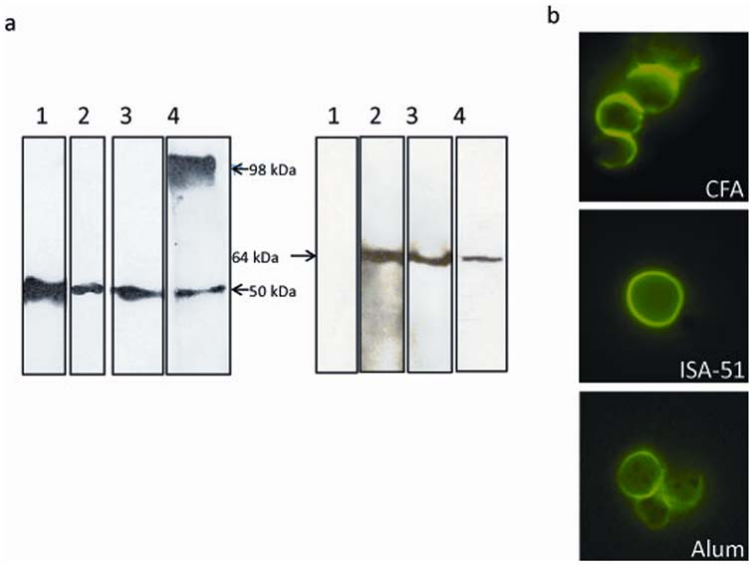
Recognition of native Pfs48/45 in *P. falciparum* gametocyte extract. (a) Western blot analysis with non-reduced (left panel) or reduced (right panel) *P. falciparum* gametocyte extract against serum of individual mouse immunized with either CFA or ISA-51 or alum formulation. Stage V gametocyte extract was run either in non-reduced or reduced (10 mM 2-mercaptothanol) form in SDS-PAGE and transferred to nitrocellulose membrane. Mice sera were allowed to react at 1∶1000 dilution for 1 h at 22°C. HRP-conjugated anti-mouse IgG at 1∶10000 dilution was used as detection antibody and was developed using ECL substrate. Lane 1, mAb IIC5B10; lane 2, one representative mouse serum immunized in CFA; lane 3, one representative mouse serum immunized in Montanide ISA-51; lane 4, one representative mouse serum immunized in alum formulation. The figure is assembled from separate experiments. (b) Mouse sera (1∶1000 dilution) were tested by live immunofluorescence assays as described under materials and methods.

We also tested individual mouse sera for their ability to recognize the native Pfs48/45 protein in gametocyte extracts in both reduced and non-reduced form in Western blot analysis. Polyclonal antisera against CH-rPfs48/45 recognized a combination of reduction-sensitive as well as reduction-insensitive epitopes in Pfs48/45 in the parasites. Sera from mice immunized with CFA and ISA-51 formulations recognized the native non reduced protein (48/45 kDa), however, the sera from mice immunized with alum formulation revealed recognition of 48/45 kDa protein and also a protein ∼98 kDa[Fig. 3a, left panel]. The reduced form of the native protein was recognized by all of the aforementioned sera at ∼64 kDa[Fig. 3a, right panel]. As previously demonstrated [24] the mAb IIC5B10 reacted only with nonreduced native parasite antigen. The ability of the anti-CH-rPfs48/45 sera to recognize the native form of the protein was further tested by live immunofluorescence assay (IFA) using live extracellular gametes. Figure 3b shows an example of strong gamete surface reactivity typical of that observed for sera from mice immunized with CH-rPfs48/45 in all three adjuvant groups.

To assess the functionality of the immunized sera, they were tested for transmission blocking activity in mosquitoes in membrane feeding assays (MFA) [10]. All the mice sera in the three adjuvant groups showed a strong (>98% reduction in the number of oocysts) transmission blocking activity at 1∶2 dilution [Table 1] as compared to corresponding pre-immune sera. Geometric mean oocyst numbers per midgut in the presence of pooled pre-immune sera of mice immunized in various adjuvant formulations and tested at different dilutions ranged between 10.4 and 16.7. The decrease in oocyst number/midgut by each immune sera was significant (*P*<0.02, Mann-Whitney test).

**Table 1:** MFA with sera from mice immunized with CH-rPfs48/45 formulated in CFA, Montanide ISA-51 or Alum.

	Serum dilution		
Animal no.	1∶2	1∶4	1∶8
**CFA**			
1	100.0 (0/20)	89.6 (8/20)	76.8 (18/33)
2	97.5 (14/41)	88.0 (7/16)	79.3 (24/38)
3	98.3 (7/28)	90.2 (4/15)	72.6 (16/34)
4	94.3 (12/22)	90.5 (9/26)	83.5 (21/42)
5	98.0 (9/30)	93.9 (6/20)	84.6 (15/28)
**ISA 51**			
6	96.1 (5/15)	88.0 (16/23)	76.8 (12/19)
7	98.4 (2/12)	89.9 (11/20)	74.9 (10/17)
8	96.4 (7/18)	92.5 (12/22)	69.7 (16/22)
9	96.2 (7/15)	90.6 (15/25)	72.6 (11/18)
10	95.1 (10/20)	86.2 (10/17)	79.1 (12/19)
**Alum**			
11	93.5 (11/24)	86.2 (14/30)	54.9 (21/29)
12	95.3 (9/26)	78.6 (16/24)	58.3 (14/20)
13	96.6 (12/31)	89.9 (6/15)	55.4 (15/22)
14	94.5 (7/16)	89.2 (9/17)	70.5 (16/26)
15	95.2 (5/16)	91.6 (6/16)	61.4 (16/23)

Individual mouse sera were tested for transmission blocking activity with respect to corresponding pooled pre-immune sera. Data are represented as percent transmission blocking activity (reduction in the number of oocysts per mosquito midgut). Numbers within parenthesis represent total number of infected mosquitoes/total number of mosquitoes dissected for each feed.

The transmission blocking effect was dependent upon the antibody dose as revealed by a gradual decrease with increasing sera dilutions. Sera from mice immunized with CH-rPfs48/45 vaccine in CFA and Montanide ISA-51 formulations as compared to Alum appeared to be relatively more potent blockers as apparent from the stronger transmission blocking activities at 1∶8 dilution of sera in MFA.

### Evaluation of functional immunogenicity of CH-rPfs48/45 in non human primates (Olive baboons)

Backed by strong functional immunogenicity of CH-rPfs48/45 in three different adjuvant formulations in mice, we next evaluated CH-rPfs48/45 vaccine in nonhuman primates (*Papio anubis*, Olive baboons). The vaccine trial in baboons was approved by the institutional and scientific review committee of the Institute of Primate Research with a protocol #19/10/2007.

**Figure 4:**
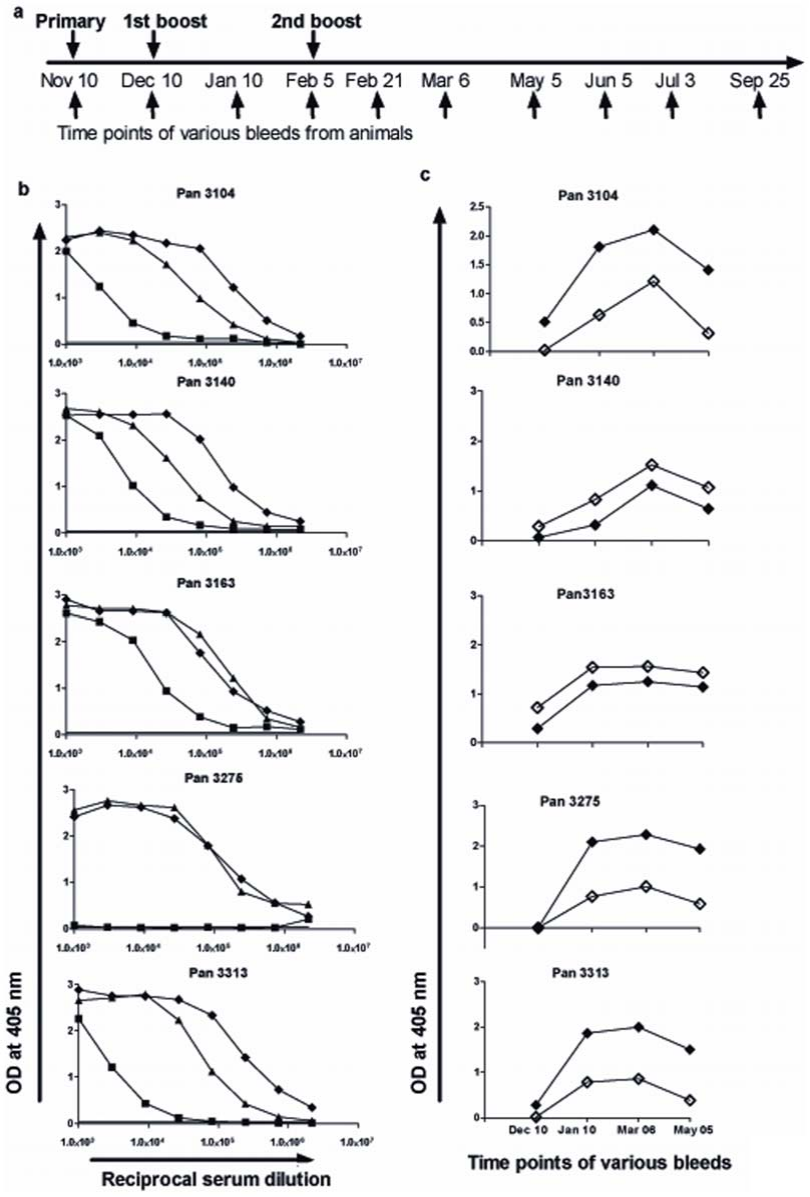
Analysis of anti-Pfs48/45 antibody production by Olive baboons (*Papio anubis*). (a) Each animal was immunized with CH-rPfs48/45 (50 µg in 0.25 ml endotoxin free PBS) formulated in Montanide ISA-51 (0.25 ml) in baboons, administered intra muscularly (quadriceps, two sites). Schedules for immunization and bleeds are indicated and sera were stored at −20°C until shipped frozen from Kenya to Baltimore for ELISA and MFA. The samples were shipped under an export permit CITES # 008101. (b) Anti-Pfs48/45 whole IgG titer at various time points analyzed by ELISA. Pre-immune +3 x SD is shown by solid horizontal lines. ELISA readings with sera dilutions, 1 month post primary immunization (filled square), 1 month post first boost (filled triangle) and 1 month post second boost (filled diamond) are shown with±SD for individual baboons (Pan 3104, Pan 3140, Pan 3163, Pan 3275, Pan 3313). (c) Distribution of anti-Pfs48/45 IgG1(solid diamonds) and IgG2 (open diamonds) subtypes in 1 month post primary immunization sera (Dec 10), 1 month post 1^st^ boost (Jan 10), 1 month post 2^nd^ boost (Mar 06), and 3 months post 2^nd^ boost (May 05). Data are presented as mean OD_405_ value±SD for individual baboon.

A group of 5 baboons (ranging 7.6 to 12.2 kg in body weight) were immunized with 50 µg of CH-rPfs48/45 in Montanide ISA-51, water-in-oil emulsion. Our choice of the adjuvant for these studies was dictated, in part, by the fact that the adjuvant has already been in use as an investigational adjuvant in clinical trials in humans [25] and that the CH-rPfs48/45 vaccine formulated in Montanide ISA-51 was strongly effective in murine immunization studies (above). The vaccine, dose, route and schedules selected were based on experience with numerous other malaria vaccine trials in nonhuman primates [26], [27], [28]. In our studies the vaccine was delivered through the intramuscular route (quadriceps, two sites) and boosted twice with the same dose of protein at 4 and 12 weeks post primary immunization [Fig. 4a].

To evaluate the immunogenicity of CH-rPfs48/45 in baboons, blood samples were collected from the immunized animals and assessed by ELISA. All, except one animal (Pan 3275), responded strongly after the primary immunization showing more than 8×10^4^ IgG titers 1 month after primary immunization. The titers increased to >1 million, 1 month after the first dose of booster, even in the case of the single primary dose unresponsive animal [Fig. 4b], reaching an antibody titer of more than 2 million following the second booster injection. We also tested the IgG subtypes with various bleeds. Three out of five animals showed predominantly IgG1 compared to IgG2, and for the other two animals, the reverse was the case [Fig. 4c]. IgG3 was totally absent from immunized sera in all the bleeds tested.

Mosquito MFA showed strong transmission blocking activity by all animals for various bleeds obtained at different time points post immunization [Table 2]. Even after primary immunization, the sera (at 1∶2 dilution) were capable of impressive transmission blocking with more than 93% average reduction in the number of oocysts in comparison to pre-immune sera of corresponding animal. Pre-immune sera from each animal were used as a measure of 100% transmission for the corresponding test sera. Pre-immune serum from one animal (Pan 3275) exhibited ∼30% reduced transmission when compared with the pre-immune sera of other four baboons. It is possible that natural infection by other *Plasmodium*-like parasites, such as *Enteropoides* and *Hepatocystis* spp might elicit partly cross-reactive and inhibitory activity. The average transmission blocking activity increased to greater than 97% in all the animals after a booster dose. In order to titrate the blocking effectiveness of immune sera from these animals, we further tested the sera obtained one month after the second boost at various dilutions (1∶4, 1∶8, 1∶16) in MFA. While exhibiting strong blocking activity at 1∶4 and 1∶8 dilutions, the sera at 1∶16 dilution were still able to reduce transmission (ranging from 74% to 86%).

**Table 2:** Transmission blocking activity of sera of different bleeds at various time points from baboons immunized with CH-rPfs48/45 in Montanide ISA-51.

Animals	Bleeds [% transmission blocking * (infected/total mosquito)]				
	12/10/07	01/10/08	02/21/08	03/06/08	05/05/08
Pan 3104	92.8 (14/27)	95.7 (11/25)	98.7 (6/22)	97.7 (7/27)	98.8 (4/19)
Pan 3140	94.4 (14/21)	97.3 (12/28)	98.1 (6/24)	98.4 (7/23)	97.8 (7/18)
Pan 3163	98.1 (5/19)	98.5 (5/18)	97.3 (9/27)	97.3 (7/20)	97.4 (11/24)
Pan 3275	88.1 (12/20)	95.3 (11/25)	96.2 (10/28)	96.2 (9/23)	97.6 (5/22)
Pan 3313	91.8 (13/25)	97.0 (9/24)	94.7 (13/27)	97.8 (8/20)	95.3 (12/24)

* *P*<0.0001 (Mann-Whitney test).

MFA was done with sera collected on 12/10/2007 (1 mo post primary immunization), 01/10/2008 (1 mo post 1^st^ boost, 02/21/2008 (15 d post 2^nd^ boost), 03/06/2008 (1 mo post 2^nd^ boost), and 05/05/2008 (3 mo post 2^nd^ boost). The geometric mean of oocyst numbers/midgut in the presence of individual pre-immune sera were 22.24 (Pan 3104), 18.43 (Pan 3140), 22.1 (Pan 3163), 6.31 (Pan 3275), and 19.7 (Pan 3313), respectively. Numbers within parenthesis represent total number of infected mosquitoes/total number of mosquitoes dissected for each feed.

**Figure 5:**
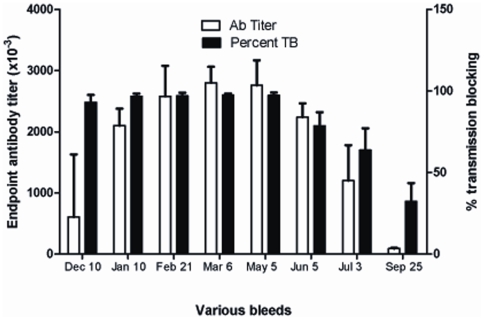
Follow up of immune responses elicited by CH-rPfs48/45 in baboons. Analysis of anti-CH-rPfs48/45 IgG titers (open bars) and percent transmission blocking activity (closed bars) upto 7 months post second boost in baboons. Results show mean antibody titer and mean transmission blocking activity of 5 baboons +95% CI.

After the second booster dose, all five animals were bled at monthly intervals and sera analyzed for antibody titers by ELISA and functional transmission blocking activity in MFA. As shown in figure 5, high antibody titers and high transmission blocking activities were maintained for more than 7 months suggesting further long lasting nature of immune responses elicited by CH-rPfs48/45 in nonhuman primates.

## Discussion

Together our data report for the very first time the expression of full length soluble recombinant Pfs48/45 protein in proper conformation with high yield following application of the codon harmonization approach for recoding gene sequences. The recombinant protein (designated CH-rPfs48/45) showed remarkable immunogenicity and functional activity, i.e. transmission blocking activity in mice immunized in the three adjuvant formulations tested. Further significance of our results is reflected by evidence from pre-clinical vaccine testing in nonhuman primates. The vaccine revealed potent immunogenicity and effective blocking activity even after a single immunization which became much stronger (approaching 100% reduction) after a booster immunization. Ease of expression and purification of protein at high yields, conformational folding and strong immunogenicity of CH-rPfs48/45 in mice and non human primates provide a much needed rationale for moving ahead with the development of malaria TBV based on Pfs48/45 antigen. Mosquito infection (oocyst load and percent infected mosquitoes) in the presence of 4/5 pre-immune sera were comparable to those obtained with normal human serum negative control.

Expression of *P. falciparum* proteins in heterologous hosts is especially challenging due to high A/T content within protein coding genes (>80%). All previous attempts to express properly folded full length recombinant Pfs48/45 have remained unsuccessful. In this study we employed a relatively recent approach of codon harmonization to express Pfs48/45 in *E. coli* in a functionally correct conformation. Whether similar adjustments for relative codon usage can be employed for expressing other transmission blocking antigens. i.e. Pfs230 and Pfs25 remains to be seen. Another strict requirement for the development of a transmission blocking vaccine is the production of proteins in correctly folded conformation. Not only did we find a robust expression of Pfs48/45 after codon harmonization of the coding sequence, the purified protein was found to be in correct conformation as revealed by Western blot analysis using monoclonal antibodies directed against reduction-sensitive conformational epitopes. Curiously, the recombinant Pfs48/45 was recognized by the same antibodies even after treatment with reducing agents, suggesting that the target epitopes in the expressed proteins are stable and not susceptible to reduction.

Further development of a transmission blocking vaccine based on Pfs48/45 is supported by the observation that the purified protein exhibited very strong and longer lasting functional immunogenicity in baboons. The antibodies elicited by vaccination continued to effectively suppress, even 5–6 months after the last booster immunization, infectivity (both oocyst burden and percentage of mosquitoes infected) of *P. falciparum* gametocytes in *Anopheles* mosquitoes. Previous studies have shown that Pfs48/45 proteins normally expressed in the erythrocytic gametocyte stage of the parasite is also a target of partially effective natural immune response. It has been hypothesized that a vaccine induced response could be further boosted during natural infection and thus help in maintaining higher antibody levels in the vaccinated people. Strongly backed by our success, efforts are currently underway to produce GMP grade Pfs48/45 for the development of a vaccine formulation for clinical trial in humans.

A sustained transmission reduction will be essential to meet the challenge of elimination of the malaria disease and perhaps even complete eradication from various endemic countries, as contemplated in the goals of the global malaria action plan of the Roll Back Malaria Partnership [29], [30], [31]. A vaccine targeting the process of malaria transmission could greatly facilitate achieving such a goal of progressive elimination of malaria.

## Materials and Methods

### Cloning, expression and purification of CH-rPfs48/45

The codon harmonized sequence of Pfs48/45 containing 6x Histidines at N-terminal end was synthesized by Retrogen Inc. and cloned into the expression vector pET(K−) in *E. coli* BL21 (DE3) competent cells (Invitrogen Corp.). The cells containing pET(K^−^)Pfs48/45 were grown overnight at 30°C in LB media containing 1% glucose and 50 µg/ml Kanamycin. The overnight culture was diluted 100 fold into 1 l culture in above mentioned media and grown at 30°C with agitation until the OD_600_ of the culture reached 1.00. The cells were then induced with 0.1 mM of IPTG and grown for 3 h at 30°C. The cells were then harvested and centrifuged at 3800 x g at 4°C for 20 min. The pellet was kept at −80°C for further processing. The frozen pellet was resuspended in 1x PBS (pH 7.4) at a pellet to buffer ration of 1∶10 (w/v) and was lysed by microfluidization (Model M110Y, Microfluidics). The lysate was centrifuged at 24000 x g for 45 min at 4°C. The lysate pellet was resuspended in 1x PBS containing 1% Tween-80 (final v/v), extracted for 30 min at 22°C and centrifuged at 24000 x g for 30 min at 4°C. The pellet was resuspended in 1x PBS containing 0.5% Sodium Lauroyl Sarcosine (sarcosyl) (final v/v), extracted and centrifuged as before. The supernatant was then passed through Ni-NTA agarose column (QIAGEN) according to manufacturer's protocol. The protein was eluted as 1 ml fractions with 1 M imidazole in 1x PBS as elution buffer. The protein was dialyzed against 1x PBS (pH 7.4) containing 10% glycerol and 0.2% Tween-80. Finally, the protein content was estimated using BCA^TM^ Protein Assay kit (Pierce) and the endotoxin level in the protein was measured using QCL-1000 Endpoint chromogenic LAL assay kit (Lonza).

### Characterization of CH-rPfs48/45

The CH-rPfs48/45 was characterized by Western blot analysis. Briefly, samples from each purification step described above were run on SDS-PAGE and transferred to nitrocellulose membrane (Bio-Rad). The membrane was blocked overnight with 1x PBS containing 5% non-fat dry milk and 0.1% Tween-20 (blocking buffer) at 4°C. Following blocking, the membrane was washed with 1x PBS containing 0.1% Tween-20 (wash buffer) and incubated with either 6xHis mAb (Clontech) at 1∶1000 dilution [Fig. 1d, e] or IIC5B10 mAb (MR4) at 1∶5000 dilution [Fig. 1f] for 1 h at 22°C. The membrane was washed 4x in wash buffer for 30 min at 22°C and incubated with HRP-conjugated anti-mouse IgG mAb (GE Healthcare) at 1∶10000 dilution in blocking buffer for 1 h at 22°C. This was followed by washing 4 x with wash buffer and ECL Plus chemiluminescent substrate (GE Healthcare) was used as detection reagent.

### Immunization of mice

Groups (n = 5) of female BALB/c mice were immunized with 10 µg of CH-rPfs48/45 emulsified in either Complete Freund's Adjuvant (CFA) (Sigma) or Montanide ISA-51 (SEPPIC) or mixed in aluminium hydroxide (Alhydrogel, Brenntag) adjuvant through the intraperitoneal route. The mice immunized in CFA were boosted at 4 week intervals twice with the same quantity of protein in incomplete Freund's adjuvant. Mice in the other adjuvant groups were boosted at 4 week intervals thrice in Montanide ISA-51 or Alum, respectively. Groups of control mice were immunized with adjuvant formulations only. Blood was collected on day 0 (Pre-immune sera) and 4 weeks after primary immunization and 2 weeks post each boost for analysis of anti-Pfs48/45 IgG titer.

### Immunization of Olive baboons (*Papio anubis*)

The vaccine trial in baboons was approved by the institutional and scientific review committee of the Institute of Primate Research with a protocol #10/10/2007. Because these animals were trapped from their wild habitats, they were quarantined for 3 months and screened for the presence of any worms and protozoan parasites and successfully treated appropriately, if found infected, prior to initiating vaccination. Moreover, animals were also screened by three intradermal tuberculin tests and found to be negative for mycobacterial infections. Detailed hematological tests were also administered on all five animals during their quarantine period, just prior to and at the termination of the vaccine trial, and were certified to be in excellent health at all time points with no observable trial-related effects. A group of five baboons (ranging 7.6 to 12.2 kg in body weight) were immunized with 50 µg of CH-rPfs48/45 in Montanide ISA-51, water-in-oil emulsion. The animals were sedated with ketamine (10 mg/kg) for immunization and blood collection as per the schedule described in Figure 4a.

### ELISA

To assess the immunogenicity of CH-rPfs48/45, ELISA was done. Briefly, Immulon-2 plates were coated with 1.5 µg/ml CH-rPfs48/45 in carbonate-bicarbonate buffer (pH 9.6) overnight at 4°C, blocked with 5% milk in PBS and incubated with various dilutions of sera at 37°C for 1 h. The plates were washed 5 times in PBS-0.05% Tween-20 (PBST) followed by further incubation with 1∶10000 dilution of horseradish peroxidase conjugated anti-mouse IgG antibody for 1 hour at 37°C. After washing in PBST, wells were developed using ABTS substrate (Kirkegaard & Perry Laboratories Inc.) for 20 min at 22°C and read at 405 nm in the ELISA reader. Anti-Pfs48/45 whole IgG end point titers were calculated from the highest group mean reciprocal serum dilution greater than the mean plus 3 standard deviations OD reading of pooled pre-immune sera in each assay. For IgG subtype analysis, mouse sera were tested at a single 1∶ 1000 dilution. Various isotype- specific secondary antibodies used were anti-mouse IgG1, IgG2a, IgG2b and IgG3 from Kirkegaard & Perry Laboratories Inc.

The ELISA with baboon sera was done following a similar protocol and endpoint titers were calculated using the same criteria. The secondary antibody was anti-human IgG1, IgG2, and IgG3 conjugated to peroxidase (The Binding Site, Birmingham, UK) and used at 1∶ 5000 dilution. Various sera were tested at 1∶5000 dilution for IgG subclass analysis.

### Parasite

*Plasmodium falciparum* NF54 parasites were maintained using normal red blood cells and normal human serum (O+ve blood group) as s described [32]. Stage V gametocytes were used in live IFA studies and membrane feeding assays. To extract gametocytes for Western blot analysis, the gametocyte culture was centrifuged at 1000 x g for 5 min at 22°C. The RBC was lysed with 0.15% saponin in PBS for 5 min at 22°C. The gametocytes were collected after centrifugation at 1800 x g for 5 min and washed thrice in 1x PBS. The gametocytes were resuspended in 25 mM Tris-Cl (pH 7.5) containing 150 mM NaCl and 1x protease inhibitor cocktail and kept frozen at -70°C till use.

### Live IFA

The gametocytes were harvested from 19 day culture and gametes were produced by incubating the gametocytes in exflagellation buffer and purified by discontinuous Nycodenz gradient centrifugation as described previously [33]. Extracellular gametes were incubated at 4°C with 1∶100 to 1∶1000 dilution of immune murine sera for 60 min. Parasites were washed 3 times with 1% BSA in PBS followed by incubation with FITC-anti mouse antisera (Alexa fluor 488), 1∶500 dilution at 4°C for 60 minutes. After washing, cells were examined by upright fluorescent Nikon E800 microscope (Japan) at 100X magnification.

### Membrane Feeding Assay

To test the transmission blocking activity, the murine and baboon immune sera were mixed with cultured *P. falciparum* (NF54) stage V gametocytes, normal red blood cells and normal human sera (donor blood group: O+) and fed to *Anopheles gambiae* (starved for 5–6 hours) mosquitoes through water jacketed glass membrane (stretched parafilm) feeders [10], [33] Briefly, washed human red blood cells and cultures containing stage V gametocytes were resuspended to 66% hematocrit and 0.3% gametocytemia in normal human serum and maintained throughout at 37°C. Fifty microliters of various test sera (appropriately diluted in normal human sera) were mixed with 150 µl of resuspended gametocyte mix and quickly added to individual membrane feeders placed on top of cups containing starved mosquitoes. The mosquitoes were allowed to engorge for 15 min. The unfed mosquitoes were separated within 1 h of feeding and blood fed mosquitoes (typically 25–30 per cup) were maintained in the insectary at 26°C at 80% relative humidity. In certain cases the total number of blood fed mosquites was less than 25 due to poor feeding. Moreover, a small number of blood fed mosquites (less than 5%) did not survive 9–10 days incubation period prior to dissection. Midguts were dissected 9–10 days after blood meal for enumeration of oocysts after staining with 1% mercurochrome. Transmission blocking activity of individual sera was calculated as percentage of reduction in oocyst number per midgut with test sera in comparison with pooled pre-immune sera (pre-immune murine or baboon sera was taken as allowing 100% transmission).
